# Virulence Potential and Genomic Mapping of the Worldwide Clone *Escherichia coli* ST131

**DOI:** 10.1371/journal.pone.0034294

**Published:** 2012-03-23

**Authors:** Jean-Philippe Lavigne, Annette C. Vergunst, Lucie Goret, Albert Sotto, Christophe Combescure, Jorge Blanco, David O'Callaghan, Marie-Hélène Nicolas-Chanoine

**Affiliations:** 1 Institut National de la Santé et de la Recherche Médicale, U1047, UFR Médecine, Université Montpellier 1, Nîmes, France; 2 Université Montpellier 1, UFR Médecine, Nîmes, France; 3 Laboratoire de Bactériologie, Virologie, Parasitologie, CHU Carémeau, Nîmes, France; 4 EA2992, Université Montpellier 1, UFR Médecine, Nîmes, France; 5 Division of Clinical Epidemiology, Geneva University Hospital, University of Geneva, Geneva, Switzerland; 6 Laboratorio de Referencia de E. coli, Departamento de Microbioloxia e Parasitolxia, Facultade de Veterinaria, Universidade de Santiago de Compostela, Lugo, Spain; 7 Service de Microbiologie, AP-HP Beaujon, Clichy, France; 8 Faculté de Médecine D. Diderot, Université Paris 7, Paris, France; 9 Institut National de la Santé et de la Recherche Médicale, U773, Centre de Recherche Biomédicale Bichat-Beaujon (CRB3), Université Paris 7, Faculté de Médecine D. Diderot, Paris, France; Université d'Auvergne Clermont 1, France

## Abstract

Recently, the worldwide propagation of clonal CTX-M-15-producing *Escherichia coli* isolates, namely ST131 and O25b:H4, has been reported. Like the majority of extra-intestinal pathogenic *E. coli* isolates, the pandemic clone ST131 belongs to phylogenetic group B2, and has recently been shown to be highly virulent in a mouse model, even though it lacks several genes encoding key virulence factors (Pap, Cnf1 and HlyA). Using two animal models, *Caenorhabditis elegans* and zebrafish embryos, we assessed the virulence of three *E. coli* ST131 strains (2 CTX-M-15- producing urine and 1 non-ESBL-producing faecal isolate), comparing them with five non-ST131 B2 and a group A uropathogenic *E. coli* (UPEC). In *C. elegans*, the three ST131 strains showed intermediate virulence between the non virulent group A isolate and the virulent non-ST131 B2 strains. In zebrafish, the CTX-M-15-producing ST131 UPEC isolates were also less virulent than the non-ST131 B2 strains, suggesting that the production of CTX-M-15 is not correlated with enhanced virulence. Amongst the non-ST131 B2 group isolates, variation in pathogenic potential in zebrafish embryos was observed ranging from intermediate to highly virulent. Interestingly, the ST131 strains were equally persistent in surviving embryos as the non-ST131-group B2 strains, suggesting similar mechanisms may account for development of persistent infection. Optical maps of the genome of the ST131 strains were compared with those of 24 reference *E. coli* strains. Although small differences were seen within the ST131 strains, the tree built on the optical maps showed that these strains belonged to a specific cluster (86% similarity) with only 45% similarity with the other group B2 strains and 25% with strains of group A and D. Thus, the ST131 clone has a genetic composition that differs from other group B2 strains, and appears to be less virulent than previously suspected.

## Introduction

*Escherichia coli* is the most common bacterial species in urinary tract infections (UTIs). These infections account for more than 11 million physician visits annually in the United States and 4 million in France [1], [2]. Uncomplicated community-onset UTIs are generally empirically treated but the risk of treatment failure is growing due to the increased antibiotic resistance within uropathogenic *E. coli* (UPEC) [3]. The recent emergence and rapid worldwide dissemination of UPEC, resistant to extended-spectrum cephalosporins due to the production of CTX-M extended spectrum β-lactamases (ESBL), has clearly shown that *E. coli* antibiotic resistance is currently a real public health concern [4]–[8].

Among the CTX-M β-lactamases, CTX-M-15 has been shown to be the most frequent and, recently, we have reported the worldwide propagation of clonal CTX-M-15-producing *E. coli* isolates distinguishable by their sequence type (ST) and serotype, namely ST131 and O25b:H4 [9], [10]. Since, the first description of clone ST131 as producer of CTX-M-15, several studies have shown that it has been a non-ESBL-producing and frequently fluoroquinole-resistant agent causing a high proportion of UTI for several years [11]–[13]. Interestingly, this clone also was shown to be the faecal dominant *E. coli* population in some healthy subjects [14]. Like the majority of extra-intestinal pathogenic *E. coli* (ExPEC) isolates, the pandemic clone ST131 belongs to phylogenetic group B2 [9], [10].

The great majority of *E. coli* strains belonging to genetic group B2 are highly virulent in a mouse model of extraintestinal infection with the exception of strain ED1a, a commensal strain belonging to the B2 subgroup VIII [15], [16]. Despite the absence of several genes encoding classical virulence factors (*pap*, *cnf1* and *hlyA*) [9], [17], [18], Clermont and colleagues suggested that the ST131 clone is virulent since, like other B2 isolates, it killed 100% of the animals in a mouse model of systemic infection [17].

To get more insight into the virulence potential of clone ST131, we used an optical mapping method to precisely characterize the genome of this clone and assessed its virulence in two other infection models, *Caenorhabditis elegans* and zebrafish embryos. The zebrafish embryo model has recently been reported to be a valuable model to resolve diverse virulence phenotypes of closely related ExPEC strains [18]. We compared the virulence of three ST131 strains from different sources (urine, stool) with that of two group B2, one group A UPEC, and three B2 reference strains. In contrast to earlier reports in mice [17], our data show that the ST131 strains are not highly virulent in nematodes and zebrafish embryos, and that the presence of the CTX-M-15 encoding plasmid is not correlated with high virulence levels. This finding is in line with the absence of several classical virulence factors such as HlyA and Cnf1, and suggests an absence of production of other important toxins or virulence factors that could cause high virulence in these models.

## Results

### Virulence potential assessed by using the *C. elegans* model

Our recent work has suggested that not all strains carrying plasmids with *bla*_CTX-M-15_ are virulent [19]. We were therefore interested in determining the virulence of the *bla*_CTX-M-15_-carrying clone ST131, generally thought to be virulent due to its B2 phylotype. The strains tested included 3 ST131 group B2 strains (MECB5, FV7563 and S250), 2 non-ST131 group B2 strains (NEC20 and NECS81153) and a group A isolate (NEC3) (Table 1). As reference strains, we used two archetypal ExPEC isolates, non-ST131 group B2 strains CFT073 and 536 [20] that have been shown to be highly virulent in mouse [15], [21] and zebrafish embryo models [18], and strain ED1a, a commensal group B2 strain, with low virulence in mice [16].

**Table 1:** Main characteristics of the *E. coli* clinical isolates used in this study.

Strain	Source	Serogroup	Phylogroup	ST	ESBL	Virulence traits	Reference
**MECB5**	Urine (cystitis)	O25b:H4	B2	131	CTX-M-15	*iha, fimH, sat, fyuA, iutA, kpsM II*, K5 *kps* variant, *usp, traT, ompT, iss, malX, irp2*	[6], [9]
**FV7563**	Urine (cystitis)	O25b:H4	B2	131	CTX-M-15	*afa/draBC, iha, fimH, sat, fyuA, iutA, kpsM II*, K2 *kps variant, usp, traT, ompT, malX, irp2*	[9], [36]
**S250**	Stools of a healthy subject	O25b:H4	B2	131	none	*fimH, fyuA, iutA, usp,iss, ompT, malX, irp2*	[14]
**NEC20**	Urine (cystitis)	O6:H7	B2	127	TEM-24	*fimH, hlyF, sat, iroN, fyuA, iutA, traT, ompT, malX, irp2, cnf1*	[19]
**NECS81153**	Urine (cystitis)	O6:H7	B2	73	none	*papGIII, papA, papC, papE, iha, fimH, hlyF, sat, iroN, fyuA, iutA, kpsM II*, K2 *kps* variant, *traT, ompT, malX, irp2, cnf1, sfa*	[19]
**NEC3**	Urine (cystitis)	O9:H4	A	10	CTX-M-15	*fimH, iutA, traT, ompT, malX*	[19]
**CFT073**	Blood	O6	B2	ST73	none	*fimH, sat, iutA, KpsM II, ompT, malX, hlyA, papa, papE, sfa/foc, iroN, aer, papC, papG, hly, fyuA, irp2*	[20]
**536**	Urine (UTI)	O6	B2	ST92	none	*fimH, KpsM II, hlyA, papA, papE, sfa/foc, iroN, papC, papG, hly, hra, fyuA, irp2*	[20]
**ED1a**	Faeces, (commensal)	O81	B2	ST452	none	*iha, fimH, sat, iutA, KpsM II, usp, traT, ompT, malX, hlyA, aer, fyuA, irp2*	[16]

The life span of *C. elegans* feeding on NEC3 (group A) was similar to that when avirulent *E. coli* OP50 was used as food (LT_50_ = 7.0±0.5 vs 7.5±0.3, respectively; p = not significant), indicating that strain NEC3 is not virulent for nematodes. Nematodes died faster when fed on any of the other strains showing that they were all virulent (p<0.001; Table 2). However, the commensal strain ED1a and the three ST131 strains were significantly less virulent for the nematodes than the non-ST131 group B2 strains (NEC20, NECS81153, CFT073 and 536) (LT_50_ = 4.1–4.7±1.1 vs 5.2–5.6±1.0; p<0.001; Table 2). The differences in virulence were not due to differences in the survival and proliferation of strains within the nematode intestine, since the intestine colonization by the different strains, measured after 72 h feeding, was not significantly different (approximately 10^5^ CFU/worm; Table 3). These data clearly show that the ST131 group B2 strains have virulence levels in nematodes similar to that of the commensal strain ED1a and that they are significantly less virulent than the non-ST131 group B2 strains. Moreover the group A cystitis isolate NEC3 was significantly less virulent than the ST131 strains and the commensal strain ED1a.

**Table 2:** 50% Lethal Time (LT50) of *Caenorhabditis elegans* infected by the *E. coli* ST131 strains and non-ST131 strains.

Strain	Source	Characteristics of strain	LT50 in days (CI95% inf-sup)	p MECB5 vs other strains
MECB5	Urine (cystitis)	B2, ST131, CTX-M-15	5.2 (4.9–5.6)	-
FV7563	Urine (cystitis)	B2, ST131, CTX-M-15	5.6 (5.3–5.9)	NS
S250	Stools of a healthy subject	B2, ST131	5.5 (5.1–5.8)	NS
NECS81153	Urine (cystitis)	B2, ST73	4.1 (3.8–4.4)	<0.001
NEC20	Urine (cystitis)	B2, ST69, TEM-24	4.7 (4.4–4.9)	<0.001
NEC3	Urine (cystitis)	A, CTX-M-15	7.0 (6.5–7.4)	<0.001
CFT073	Blood	B2, ST73	4.1 (3.7–4.3)	<0.001
536	Urine (UTI)	B2, ST92	4.3 (4.0–4.5)	<0.001
ED1a	Faeces, (commensal)	B2, ST452	5.6 (5.2–5.8)	NS
OP50	Laboratory	Avirulent, nematode food.	7.5 (7.2–7.8)	<0.001

The results are representative of at least five independent trials for each group of strains. p: Pairwise comparison using a log rank tests. NS: non significant.

**Table 3:** Evaluation of CFU in the *C. elegans* digestive tract obtained from 3 experiments for each strain.

Strain	Source	Characteristics	Median CFU[range]/nematode after 72 h
MECB5	Urine (cystitis)	B2, ST131, CTX-M-15	5.5×10^5^ [4.8–6.1 10^5^]
FV7563	Urine (cystitis)	B2, ST131, CTX-M-15	4.9×10^5^ [4.2–5.6 10^5^]
S250	Stools of a healthy subject	B2, ST131	6.1×10^5^ [4.9–6.6 10^5^]
NECS81153	Urine (cystitis)	B2, ST73	4.7×10^5^ [3.2–6.3 10^5^]
NEC20	Urine (cystitis)	B2, ST69, TEM-24	5.0×10^5^ [4.8–5.2 10^5^]
NEC3	Urine (cystitis)	A, CTX-M-15	2.2×10^5^ [1.0–3.4 10^5^]
CFT073	Blood	B2, ST73	3.3×10^5^ [2.5–4.0 10^5^]
536	Urine (UTI)	B2, ST92	4.1×10^5^ [3.5–4.7 10^5^]
ED1a	Faeces, (commensal)	B2, ST452	2.9×10^5^ [2.5–3.4 10^5^]
OP50	Laboratory	Nematode food, avirulent	4.2×10^5^ [2.9–5.4 10^5^]

### Virulence in the zebrafish embryo model

Based on the results obtained in *C. elegans*, we investigated whether the ST131 strains also had lower virulence in zebrafish embryos. We analysed the virulence of the same panel of strains by micro injecting bacteria (on average 1.10^3^–2.10^3^ CFU of each strain per embryo) in the blood circulation of 30 hours post fertilization (hpf) embryos.

**Figure 1:**
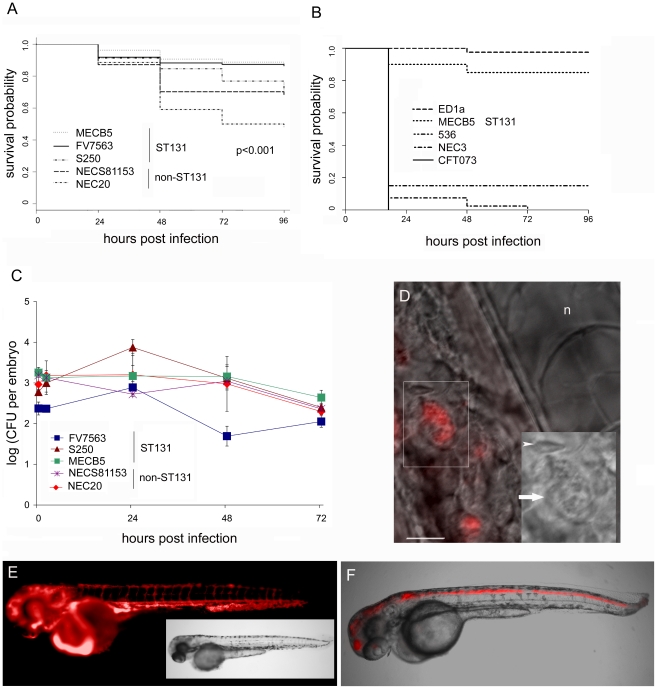
Virulence of ST131 B2 group *Escherichia coli* strains in zebrafish embryos compared to other non-ST131 B2 group strains. A. Survival probability of zebrafish embryos following infection with three ST131 strains and two non-ST131 strains. Thirty hour post fertilization (hpf) embryos were microinjected with an average inoculum size of 1.9×10^3^.CFU. The data represent the average of 5 independent experiments (n = 20 for each strain per experiment). NEC20 and NECS81153 were analysed twice. A Mantel-Cox test was performed (between strains p<0.001). NECS81153 and NEC20 did not differ significantly (p = 0.08) from each other in virulence. The non-ST131 group B2 strains NEC20 and NECS81153 induced a significantly higher rate of embryo mortality than the two ST131 strains MECB5 (p<0.001) and FV7563 (p = 0.002). MECB5 was significantly less virulent than S250 (p<0.001), but did not differ from FV7563 (p = 0.73). Although S250 and NECS81153 did not differ in virulence (p = 0.82), S250 was less virulent than NEC20 (p = 0.009). In this model, NEC20 was significantly (p<0.05) more virulent than all the other group B2 strains except for NECS81153(p = 0.08). B. Survival probability of zebrafish embryos following injection of ST131 strain MECB5 compared with group A strain NEC3, and non-ST131 group B2 control strains CFT073, 536 and ED1a. Thirty hour post fertilization (hpf) embryos were microinjected with an average inoculum size of 1.4×10^3^.CFU. The data represent the average of 2 independent experiments (n = 20 for each strain per experiment) in this context, but each strain was analysed at least 5 times. A Mantel-Cox test was performed. MECB5 was significantly less virulent than the strains CFT073, 536 and NEC3 (p<0.0001). ED1a was significantly less virulent than MECB5 (p = 0.047). Within the group of fast killing strains, strain 536 was less virulent than CFT073 (p = 0.011) and NEC3 (p = 0.020), whereas NEC3 and CFT073 were not significantly different (p = 0.079). C. Infection kinetics after microinjection with the following inocula: FV7563 (300 CFU), S250 (520 CFU), MECB5 (1760 CFU), NECS81153 (2200 CFU) and NEC20 (880 CFU). Bacterial counts were performed in 5 individual embryos per time point per strain (geometric mean ± SEM, n = 5), however, dead embryos were not included in the counts as explained in the text. Shown is a typical experiment. The experiment was repeated 5 times. D. Bright field and fluorescence overlay of a NEC20 infected embryo, 48 hpi, showing a close up of bacteria in a phagocytic cell. Inset shows an enlargement of the phagocytic cell (arrow). n = notochord, arrowhead marks an erythrocyte. E. Fluorescent image of an embryo, 24 hpi, infected with NEC20-DSRed showing bacteria have multiplied in the vasculature. A non-infected control embryo does not show any fluorescence with similar camera settings (not shown). Inset: corresponding bright field image. F. Embryo, 24 hpi, microinjected with MECB5-DSRed. Fluorescence and bright field overlay image of central nervous system infection.

In several independent experiments, we first compared mortality rates over a 96 hour period. The ST131 UTI strains (MECB5 and FV7563) were the least virulent and killed only few embryos in contrast to the two non-ST131 strains (NEC20 and NECS81153) (Figure 1A; see legend for statistical significance). Although ST131 strain S250 (a faecal isolate) caused less killing than strain NEC20, it was slightly more virulent than the other two ST131 strains, and was as virulent as strain NECS81153.

In a separate set of experiments, we compared the reference strains 536 and CFT073 (non-ST131 B2), NEC3 (group A) and ED1a (commensal B2) with the previously tested ST131 group B2 strain MECB5. To our surprise, while ED1a was not virulent for zebrafish embryos, NEC3, which was avirulent in the nematode model and lacks any classical virulence factors, was lethal for zebrafish embryos killing almost all embryos within 17 hours post infection (hpi) (Figure 1B). In fact, NEC3 was much more virulent than non-ST131 group B2 strain NEC20, which was the most virulent strain in the first set of experiments (Figure 1A). The control strains 536 and CFT073, both non-ST131 group B2 strains, were also lethal for the embryos and much more virulent than ST131 strain MECB5 (P<0.0001; Figure 1B). Interestingly, although the non-ST131 strains NEC20 and NECS81153 tested in the first set of experiments were more virulent than the ST131 strains (Figure 1A), they were not as lethal for the embryos as the strains 536 and CFT073 (Figure 1B). Together, these findings reveal variation in the pathogenic potential between different group B2 strains ranging from low virulence (ST131 strains MECB5 and FV7563, and non-STS131 strain ED1a), through intermediate virulence (ST131 strain S250, non-ST131 strains NEC20 and NECS81153) to lethal (non-ST131 strains CFT073 and 536), and show the promise of this model to study differences in virulence between UPEC isolates. Most importantly, the results are in agreement with the results obtained in nematodes, namely that the ST131 strains are not as virulent as other non-ST131 group B2 strains.

In addition to following mortality, we determined bacterial numbers in individual live embryos sampled at regular intervals over a period of 72 h. For all strains analyzed, dead and moribund embryos contained between 10^5^ to 10^7^ CFU (data not shown), whereas the number of bacteria was several orders of magnitude lower, but constant, in embryos that survived during the study period (Figure 1C; ED1a not shown; NEC3, CFT073 and 536 killed embryos rapidly and were not included in the kinetics study). This indicated that all (non-lethal) group B2 strains were equally persistent in embryos that were able to control the infection, in contrast to avirulent *E. coli* K12 (DH5α) which was phagocytosed and degraded within 5 hpi when injected with similar inoculum sizes, as previously described [22].

The transparent nature of zebrafish embryos allows us to visualize the infection in real time [21]. We followed embryos infected with DSRed expressing bacteria in real time by fluorescence microscopy. In agreement with the replication kinetics and mortality data observed in this model, two classes of infected embryos were detected. In embryos that survived during the study period, the number of bacteria was low and constant. The bacteria were observed in phagocytic cells (Figure 1D), sometimes moving in the blood circulation (not shown) at later time points during infection; however, we can not exclude that bacteria present in extracellular aggregates contributed to the observed persistence. In contrast, in embryos that subsequently succumbed to the infection, the number of bacteria increased dramatically. In these fatal infections we observed bacteraemia, with DSRed-expressing bacteria filling the vasculature of the embryos (Figure 1E) for all strains. Unexpectedly, in about 10–20% of the embryos injected with the ST131 strains MECB5 and S250, and non-ST131 strains NEC 20 and NECS8115, we observed a tropism towards the central nervous system (CNS) (Figure F). We did not observe tropism for the CNS with ST131 strain FV7563, and did not analyze this phenomenon in the fast killing strains 536, NEC3, CFT073, nor in ED1a (as we were unable to introduce the DSred plasmid in this strain). CNS infections were sometimes accompanied by bacteraemia but could also be observed in the absence of massive bacterial propagation elsewhere in the embryos; in all cases, embryos in which bacteria had propagated to high levels died due to the infection.

### Optical maps analysis and phylogenetic tree

To understand why the three O25b:H4-ST131 strains were less virulent than the other group B2 strains, we used optical mapping to analyse their genome organisation. Optical maps were constructed for *Nco*I digested chromosomes and compared with *in silico* maps generated from the complete genome sequences of 26 *E. coli* strains available in GenBank (www.ncbi.nlm.nih.gov/genbank/).

**Figure 2:**
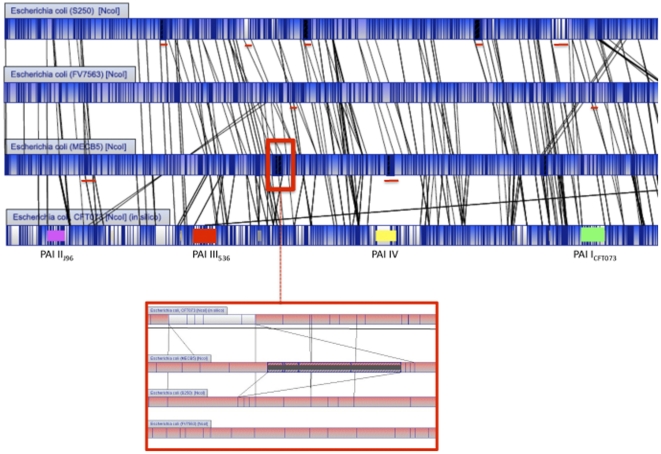
Overview of optical maps of the *E. coli* ST131 strains S250, FV7563 and MECB5, and reference UPEC CFT073. Alignment software highlights *Nco*I restricted fragment polymorphisms between strains by using white color and homologous regions by using blue color. Vertical lines in each bacterial genome represent matching *Nco*I restriction sites. PAIs present in the genome of strain CFT073 are represented by different colors: PAI I: green, PAI II: pink, PAI III: red, PAI IV: yellow. Red lines under the genome of S250, FV7563 and MECB5 indicate four insertions only observed in this strain. Red frame on the genome of strain MECB5 indicates the insertion only observed in this strain. A close up view of this region is shown under the genome optical maps.

Among the three ST131 strains, MECB5 and FV7563 were the most similar, showing 96.3% similarity. The difference was essentially due to 3 regions (Figure 2); two insertions of approximately 30 kb present in strain FV7563 and a 131 kb insertion in the MECB5 genome, which was not found in any other *E. coli* optical map or sequence. None of the insertions were found to interrupt an operon or a PAI. The 131 kb region in MECB5 was situated between genes encoding a hypothetical protein (YchH) and a ribose-phosphate pyrophospohokinase (PrsA).

Among the ST131 strains, the S250 genome showed 86.1% similarity to that of strain MECB5. Five insertions (from 20 to 50 Kb) were observed in strain S250 and not in MECB5, and two (40 and 60 Kb) in strain MECB5 and not in strain S250. Comparison with the *in silico* maps of two reference strains (UTI89 and CFT073) allowed us to localize two of the five insertions observed in strain S250. One was inserted between a gene encoding a hypothetical protein (YdiV) and a gene encoding a part of CelABC permease (CelA), and the second between a gene encoding an oxydoreductase (YdfG) and a gene encoding a starvation sensing protein (RspA), respectively. The three other insertions observed in strain 250, and two insertions in MECB5 were not inserted in PAI or operons and were specific to these strains.

Three major differences were observed when the genome maps of the three ST131 strains were compared with that of group B2 strain CFT073; in the three ST131 strains, PAI III_536_ was considerably modified (deletion of 86 Kb), leading to the absence of genes encoding adhesins (*focD*, *focH*) and siderophores (*iroB* to *iroE*). Both PAI II_J96_ and PAI I_CFT073_ were absent, explaining the lack of classical UTI virulence factors such as Hly (HlyA, HlyB, and HlyC), cyclomodulins (Cnf1) and Pap (PapG, PapX, PapF, PapE, PapK, PapJ, PapD, PapC) in the three ST131 strains. The other *pap* genes (*papD2*, *papE2*, *papF2*, *papJ2*, *papK2*, *papH2*, *papA2*, *papI2*) classically located downstream PAI I_CFT073_ were also absent in the three ST131 strains. Optical map analysis also confirmed the results obtained by PCR (Table 3) with regards to other virulence traits showing presence of the gene encoding the secreted autotransporter toxin (Sat) in strains MECB5 and FV7563, absence of regions encoding capsules in strain S250, and presence of genes encoding proteins implied in iron metabolism, namely IutA classically found in PAI I_CFT073_, Irp2 and FyuA classically found in the high pathogenicity island (HPI), and the gene *usp* encoding a uropathogenic-specific protein in the three ST131 strains. The map analysis also confirmed that *iss* gene was present in MECB5 and S250 and absent in FV7563. Moreover, optical map analysis allowed us to show the presence of additional genes encoding virulence factors involved in iron metabolism (*chu*, *hma*; iron uptake, *sit*; iron transport) in the three ST131.

**Figure 3:**
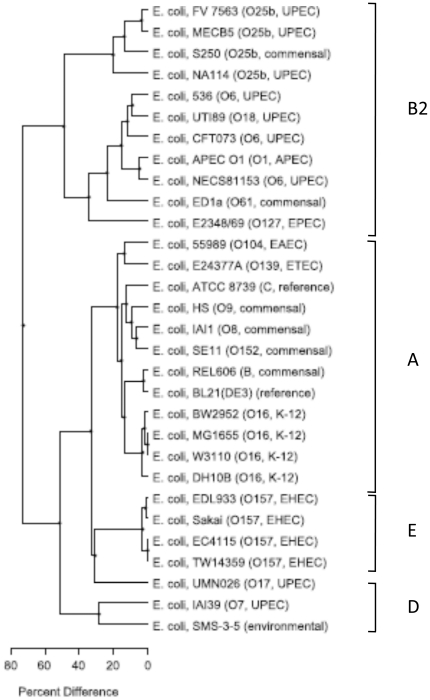
Map similarity cluster using unweighted-pair group method using arithmetic averages (UPGMA) tree of optical maps performed on different *Escherichia coli* strains. The scale indicates the percentage of genetic difference.

A phylogenetic tree was built on the basis of the optical and *in silico* maps of 28 *E. coli* strains (Figure 3). This shows that the three ST131 strains form a cluster (86% similarity) with approximately 45% similarity with the other group B2 strains and 25% with strains of groups A and D. The recently published genome of an O25b-ST131 strain (NA114) isolated in India [23] was clearly similar to the strains used in our study with 80% homology. It also showed that UPEC strain NECS81153 that was classified into the second group B2 cluster was more closely related to the classical UPEC CFT073 than to the other classical UPEC UTI89.

## Discussion

Although high virulence of the CTX-M-15-producing ST131 clone has been construed from its isolation from patients, its appendance to the B2 phylotype and the presence of genes encoding virulence factors (although key virulence factors such as Pap, HlyA and Cnf1 are missing), little work has been done to actually assess this. In a recent study Clermont et al. suggested that this clone is virulent as systemic infection of mice resulted in a lethal infection [17]. However in this study, lethality induced by clone ST131 was obtained with a very high dose of bacteria (2.10^8^ CFU). Here we used two infection models previously used to characterise the virulence of *E. coli* (*C. elegans* and zebrafish [18], [19], [24], [25]), to compare the virulence of three ST131 strains with three other UTI isolates which belong to groups A and B2 and, more importantly, to a B2 subgroup different from that of the ST131 strains. For comparison, as reference to virulence studies reported earlier, we included the two non-ST131 group B2 strains CFT073 and 536, which are highly virulent in mouse [15] and zebrafish models of infection [18], and the commensal group B2 strain ED1a that is avirulent in mice [16]. Using both models, we found that the ST131 strains containing the *bla*_CTX-M-15_ plasmid were less virulent than other non-ST131 group B2 strains lacking the plasmid (except the less virulent control ED1a), suggesting that CTX-M-15 production is not correlated with high virulence levels.

With *C. elegans*, we found that, although more virulent than NEC3 (group A), the three ST131 strains MECB5, S250 and FV7563 were less virulent than the B2 UTI isolates NEC20, NECS81153, CFT073 and 536. As the five genes, encoding the virulence factors FimH, IutA, TraT, OmpT and MalX, that were identified by PCR in the avirulent group A strain NEC3, were also present in the B2 strains (although S250 lacks *traT*), it is highly unlikely that these virulence factors would play a role in the virulence observed with the B2 strains studied. One surprising result was the lethal effect of group A strain NEC3 in zebrafish embryos, similar to the lethality of the group B2 strains CFT073 and 536. At this point, we do not know the reason for this lethal effect. It will be interesting to find out which virulence factor(s) or toxin(s) is responsible for the observed killing of zebrafish embryos by NEC3.

Using the zebrafish embryo model, we injected bacteria directly into the blood circulation and followed the course of the infection measuring bacterial multiplication, embryo survival and observing the infection in real time by fluorescence microscopy. We used younger embryos (30 vs 48 hpf) and slightly smaller inoculums than described earlier [18] for injection of zebrafish embryos with *E. coli* directly in the blood circulation. Following the infection over 96 hours allowed us to unmask differences in virulence between the B2 group strains, and in addition allowed us to show that all strains (except NEC3, CFT073 and 536, which we could not include as they killed most of the embryos within 24 hours) caused persistent infection. For comparison, non-virulent DH5α does not kill any embryos, and does not show persistent infection (data not shown and [22]). To date, we do not know the reason for this persistence. Bacteria were been detected in phagocytes at later time points in infection, but we cannot exclude the presence of extracellular bacterial aggregates. The finding that the least virulent strain ED1a can also show persistence in this model suggests other conserved mechanisms may account for development of persistent infection.

In agreement with results obtained in nematodes, the UTI strains NEC20, NECS81153, CFT073 and 536 were more virulent, and killed significantly more zebrafish embryos than the two ST131 strains MECB5 and FV7563 over a period of 4 days. Although significantly less virulent, the ST131 strains were still able to kill between 10 to 20 percent of the embryos in 4 days. These strains lack classical virulence factors (Pap, HlyA and Cnf1), and it will be interesting to determine the factors responsible for the observed low level of virulence. In addition, although less virulent than NEC20, ST131 strain S250 showed higher virulence than the two other ST131 strains, a difference that was not observed in the nematode model. This is interesting as strain S250 is a commensal strain and carries fewer genes encoding virulence factors than the two UPEC ST131 strains. In contrast to MECB5 and FV7563, S250 does not harbour the *bla*_CTX-M-15_ resistance plasmid confirming that its presence is not necessarily correlated with increased virulence in this model. A similar result was obtained with isogenic strains of *K. pneumoniae* whose virulence potential was tested with and without this plasmid in the *C. elegans* model [26]. It remains to be determined whether the presence of *bla*_CTX-M-15_ may actually reduce virulence.

Within the analysed non-ST131 group strains, we clearly distinguished strains with intermediate (NEC20 and NECS81153) and high virulence (536 and CFT073). The high virulence of CFT073 and 536 is in agreement with earlier reports in mice [15] and zebrafish [18]. Recently, Wiles *et al.* showed an essential role for zebrafish phagocytes in the control of both localized and systemic infection with extraintestinal pathogenic *E. coli* [18]. Interestingly, the authors suggested that survival and virulence of different ExPEC strains were variably dependent upon the secreted toxins HlyA and Cnf1, primarily in the evasion of phagocyte-mediated killing [18]. The Cnf1 toxin produced by UPEC has been shown to inhibit phagocyte functions by activating host Rho-family GTPases and increasing F-actin content, resulting in cytoskeletal reorganization [27]–[31]. NEC20 and NECS81153 encode Cnf1, yet these strains show intermediate virulence in our experiments. It will be interesting to analyse the contribution of Cnf1 to virulence in those genetic backgrounds.

The zebrafish model allowed us to take a closer look at the infection in real time. In agreement with the mortality assays, in embryos that succumbed to the infection bacteria were seen to multiply to high numbers in the vasculature and/or in the central nervous system prior to killing the host. Currently, we do not know which factors contribute to this tropism towards the central nervous system in zebrafish for some of the strains, and there is no clinical evidence for ExPEC strains crossing the blood/brain barrier in humans, yet our results suggest that the zebrafish will be a promising model to study invasion of the CNS by *E. coli*.

Optical mapping is a recently developed method to rapidly examine genome organisation. Comparison of the genome maps with those previously reported for UPEC strains [32] confirmed the absence of the classical extra intestinal PAIs (PAI I, PAI II and PAI III) [16], except for the high pathogenicity island (PAI IV) in the ST131 isolates. Despite of phenotypic and genomic differences, the three ST131 strains segregated into a cluster (86% similarity) in the tree built from the optical maps, that showed only 45% similarity to the other group B2 strains. This tree resembles the tree built from MLST by Le Gall *et al.* and Clermont *et al.* and confirms that clone ST131 belongs to a specific subgroup within group B2 strains [16], [17]. Our results also support the suggestion by Le Gall *et al.*, that this subgroup would be basal among the subgroups B2 [16]. The segregation into a different cluster correlated with the reduced virulence levels observed for those strains compared to the other group B2 strains in nematodes and zebrafish embryos. The cause of the intermediate virulence remains to be analyzed. We are left with the question as to why ST131, which has an intermediate level of virulence and belongs to an “old” subpopulation within group B2, has emerged recently in patients? It was identified for the first time through the analysis of multidrug resistant isolates, however a historical isolate dating from 1985 has been reported [9], [10], [33]. It is still not clear whether the source of ST131 is zooanthroponotic, however evidence is increasing to suggest that humans are the main source which occasionally spill over into animals [34], [35]. Recent studies reporting on non-ESBL-producing ST131 isolates have underlined the significant higher level of antibiotic resistance in ST131 isolates in comparison with non-ST131 isolates [33], [36].

Overall, our data suggest that (i) the virulence potential of clone ST131, which is disseminating throughout the world, is lower than that of classical UPEC (ii) the strains are not avirulent in spite of the absence of PAI I, II and III and (iii) there are strains with different level of virulence within clone ST131. *E. coli* ST131 harboring *bla*_CTX-M-15_ does not seem to be an acutely virulent pathogen; however, it can cause a persistent infection. Is finding the perfect balance between virulence and resistance the secret of clone ST131?

## Materials and Methods

### Bacterial strains and plasmids

The nine *E. coli* strains we investigated are described in Table 1; all are of the B2 phylotype, except strain NEC3 which is of the A phylotype. Three strains were O25b:H4-ST131; MECB5 and FV7563 are representatives of the worldwide clone carrying *bla*_CTX-M-15_, isolated from France and Spain, while strain S250 is a commensal gut isolate which does not carry the CTX-M-15 plasmid. For comparison, we included three UTI isolates that were not O25b:H4-ST131; NEC20 (group B2, O6:H7, TEM-24), NEC3 (group A, O9, CTX-M-15) and NECS81153 (group B2, O6), an antibiotic sensitive and virulent strain. Three other control strains were included for comparison: two virulent group B2 strains CFT073 and 596, and the avirulent strain ED1a. *E. coli* K12 DH5α was used as control in the zebrafish model and *E. coli* OP50 as food for nematodes. Bacteria were grown in Luria Bertani broth and trypticase soy broth or agar at 37°C. To allow real time analysis in zebrafish embryos, *E. coli* strains were transformed by electroporation with plasmid pIN29 that expresses the DSRed fluorescent protein [22].

### *Caenorhabditis elegans* killing model

The *C. elegans* infection assay was carried out as described by Lavigne *et al.* using the Fer-15 mutant line, which has a temperature sensitive fertility defect [37]. To synchronize the growth of worms, eggs were collected using the hypochlorite method. Overnight cultures of *E. coli* strains in nematode growth medium (NGM) were harvested, centrifuged, washed once and suspended in phosphate buffered saline solution (PBS) at pH 7.0 at a concentration of 10^5^ CFU/mL. NGM agar plates were inoculated with 10 µL of bacterial suspension and incubated at 37°C for 8–10 h. Plates were brought back to room temperature and seeded with L4 stage worms (20–30 per plate). Plates were then incubated at 25°C and scored each day for live worms under a stereomicroscope (Leica MS5). A worm was considered dead when it no longer responded to touch. Worms that died as a result of having been trapped by the wall of the plate were excluded from the analysis. At least three replicates repeated 5 times were performed for each selected strain. Lethal time 50% (LT50) corresponded to time (in days) required to kill 50% of the nematodes.

Bacterial counts in the *C. elegans* digestive tract were carried out as described by Garsin *et al.* [38]. The experiments consisted of measuring the number of bacteria within the *C. elegans* digestive tract 72 h after ingestion. Three replicates were performed for each strain.

### Zebrafish infections

*Danio rerio*, variety ‘Gold’ (purchased from Antinea SARL, Montpellier, France) were kept as described [22]. Animals were handled according to national regulations for animal welfare (Approval ID is D 30-189-4 and the study on zebrafish is approved by the ‘Direction Departementale de la Protection des Populations’). Overnight cultures of *E. coli* expressing DSRed were diluted 1/100 in Luria Bertani broth and grown with shaking for a further 3–4 hours. Dechorionated embryos were micro-injected 30 hours post fertilization (hpf) in the blood island with bacteria as described previously [22]. To follow infection kinetics, 5 embryos were collected randomly at 0, 2, 24, 48 and 72 hpi, and individually treated for bacterial enumeration as described [22]. For survival assays, embryos were maintained individually in 24 well plates in embryo medium at 29°C. At regular time points after infection, the number of dead embryos was determined visually by the absence of a heartbeat. Real time analysis was performed as described [22]. A Leica DM IRB inverted microscope equipped for bright-field, differential interference contrast (DIC), and fluorescence imaging was used. DSRed was excited using a 100-W mercury lamp, and fluorescence was detected using filter sets L5 (band pass [BP] 480/40; beam splitter [BS] 505; emission BP527/30) and N2.1 (515 to 560; BS 580; emission long pass [LP] 590), respectively. For imaging we used a Coolsnap fx (Roper Scientifique) and MetaVue software, and images were processed further using Adobe Photoshop. Embryos were transferred to glass-bottom dishes (MatTek Corp., Ashland, MA) for direct visualization using 40×, 63× and 100× oil immersion objectives.

### Optical maps

Optical maps were generated by OpGen SA (Gaithersburg, MA, USA) as described previously [39]. In situ digestion was performed with *Nco*I. Restriction map alignments between different strains were generated using OpGen's Mapviewer program. This dynamic programming algorithm finds the optimal alignment of two restriction maps according to a scoring model that incorporates fragment sizing errors, false and missing cuts, and missing small fragments. Pair-wise alignments were performed between all maps using an alignment score of 3. The optical maps of the studied strains were then compared with the *in silico* restriction maps of 26 of sequenced *E. coli* isolates whose sequence genomes were available in GenBank and transformed by using the MapSolver v.2.1.1 software (OpGen SA).

### Statistical analysis

To compare the entire survival curves in nematode killing assays, we used a Log rank test and a Cox regression analysis. In zebrafish embryos, the number of CFU per time point for each strain was expressed as the geometric mean CFU (± SEM) of five individually plated embryos. In order to perform pairwise comparison between two different strains, we used a log rank test. The analysis was carried out using SPSS 6.1.1 (SPSS Inc., Chicago, IL, USA).
